# Medical cost savings in Sakado City and worldwide achieved by preventing disease by folic acid fortification

**DOI:** 10.1111/cga.12215

**Published:** 2017-04-04

**Authors:** Yasuo Kagawa, Mami Hiraoka, Mitsuyo Kageyama, Yoshiko Kontai, Mayumi Yurimoto, Chiharu Nishijima, Kaori Sakamoto

**Affiliations:** ^1^ Department of Medical Chemistry Kagawa Nutrition University Sakado City Japan; ^2^ Shukutoku University College of Nursing and Nutrition, School of Nutrition Chiba City Japan; ^3^ Yamanashi Gakuin University Faculty of Health and Nutrition Kofu City Japan; ^4^ University of Niigata Prefecture Faculty of Human Life Studies Department of Health and Nutrition Niigata City Japan; ^5^ Yakult Honsha Co., Ltd. Tokyo Japan

**Keywords:** folate, fortification, genetic polymorphism, medical cost

## Abstract

The introduction of mandatory fortification of grains with folate in 1998 in the United States resulted in 767 fewer spina bifida cases annually and a cost saving of $603 million per year. However, far more significant medical cost savings result from preventing common diseases, including myocardial infarction, stroke, dementia and osteoporosis. A cost‐effectiveness analysis showed a gain of 266 649 quality‐adjusted life‐years and $3.6 billion saved annually, mainly due to the reduction of cardiac infarction. The recommended folate intake in Japan is 240 μg/day whereas it is 400 μg/day internationally. Our Sakado Folate Project targeted individuals with genetic polymorphism of methylenetetrahydrofolate reductase or with hyperhomocysteinemia. Using, for example, folate‐fortified rice, resulted in an increase in serum folate and a decrease in serum homocysteine in the participants, and reduced medical costs were achieved by decreasing myocardial infarction, stroke, dementia and fracture. Due to the small population of Sakado City (approximately 101 000) and small number of births (693) in 2015, a decrease in spina bifida could not be confirmed but there was a significant decrease in the number of very low birthweight infants. The genome notification of subjects was effective in motivating intake of folate, but the increase in serum folate (from 17.4 to 22.5 nmol/L, 129%) was less than that observed following compulsory folic acid fortification of cereals in the USA (from 12.1 to 30.2 nmol/L, 149.6%). Mandatory folic acid fortification is cheap in decreasing medical costs and is thus recommended in Japan.

## Introduction

The authors reviewed the medical literature reporting key studies on folic acid fortification, policy recommendations from several foreign organizations, and our own Sakado Folate Project, and drew conclusions regarding the decrease in the incidence of disease and the medical cost savings after the folate intake of the population is increased. Folate refers to a collection of “folates” that is not chemically well‐characterized and includes other members of the pteroylglutamate family. These family members are differentiated by the reduced state of the pteridine ring, a one carbon substitution at the N5 and/or N10 positions (formyl, methyl, methylene, and methenyl), and the length of the γ‐glutamyls. Polyglutamyl 5‐methyltetrahydrofolate species are the most abundant naturally occurring folate in vegetables, and 5‐formyltetrahydrofolate species are the second most abundant (Wang et al. 2010). The bioavailability of synthetic folic acid (monoglutamyl pteridine) is approximately 70% better than that of folate (mainly polyglutamyl pteridines) from dietary sources and thus the term dietary folate equivalent (DFE) is used to describe the effects of different folate molecular species (Joint FAO/WHO Expert Consultation on Human Vitamin and Mineral Requirements 1998). Folate prevents megaloblastic anemia (Chanarin 1987) and many other diseases because it is essential to the synthesis, repair (Lindahl 2013), and epigenetic regulation (Janke et al. 2015) of DNA. Folate is therefore the link between diet, metabolism, and epigenetics (Janke et al. 2015). Thus, the mechanism by which folate prevents many diseases, such as neural tube defects (NTD), has been studied at the molecular level (Ichi et al. 2012). NTDs are the second most common group of serious birth defects. Each year, 300 000 to 400 000 infants worldwide are born with NTDs. NTDs result from failure of the neural tube to close properly approximately 28 days post‐conception. Two of the most common NTDs are spina bifida and anencephaly. One of the earliest recommendations on folate intake was the Recommendations of the Clinical Teratology Committee of the Canadian College of Medical Geneticists, which stated that primary prevention of NTDs is preferable to treatment or to prenatal detection and abortion (Van Allen et al. 1993). The use of biomarkers such as homocysteine to detect folate deficiency has been described in detail by Bailey et al. (2015). There is now conclusive evidence that most NTDs can be prevented by the ingestion of folic acid near the time of conception (Joint FAO/WHO Expert Consultation on Human Vitamin and Mineral Requirements 1998). Thus, mandatory folic acid fortification of cereal products has been implemented in 82 countries. Mandatory fortification has led to medical cost savings due to the prevention not only of NTDs (Grosse et al. 2016), but also of many other diseases described in the next section (Bentley et al. 2009).

## Diseases Prevented by Folic Acid Fortification

Megaloblastic anemia (Chanarin 1987) and NTDs (Grosse et al. 2016) are well‐known conditions caused by inadequate folate intake, but folate is implicated in many other diseases. For example, moderate hyperhomocysteinemia induced by low folate status is an independent risk factor for cardiovascular disease (CVD) (Ishihara et al. 2008; Bentley et al. 2009), stroke (Yang et al. 2006; Huo et al. 2015; Li et al. 2016b), colon cancer (Bentley et al. 2009), dementia (Jernerén et al. 2015), fracture (Gjesdal et al. 2007), kidney diseases (Massy 2003) and depression (Bottiglieri 2005; Hiraoka et al. 2014). These diseases can be prevented or alleviated by increased folate intake, as shown by many epidemiological and interventional studies. The meta‐analysis of 30 randomized controlled trials involving 82 334 participants indicated a 10% lower risk of stroke and a 4% lower risk of overall CVD with folic acid supplementation (Li et al. 2016b). Folic acid supplementation provided a greater benefit for preventing CVD in participants with low plasma folate levels and without preexisting CVD, and resulted in larger decreases in homocysteine levels (Li et al. 2016b). Folate is required by the brain for the synthesis of monoamine neurotransmitters, and numerous observational studies have suggested an association between folate deficiency and depression (Bottiglieri 2005). Folate intake below the Recommended Daily Allowance at study baseline was associated with increased risk of the incidence of Mild Cognitive Impairment (MCI)/probable dementia (hazard ratio 2.0, 95% CI 1.3 to 2.9) after controlling for multiple confounders (Agnew‐Blais et al. 2015). Plasma homocysteine concentration, if only moderately elevated, is an independent risk factor for CVD and stroke (Joint FAO/WHO Expert Consultation on Human Vitamin and Mineral Requirements 1998). Increased risk has been associated with values higher than 11 mmol/L, which is well within what is generally considered to be the normal range (5–15 mmol/L) of plasma homocysteine levels (Joint FAO/WHO Expert Consultation on Human Vitamin and Mineral Requirements 1998). Randomized control tests on the prevention of CVD and dementia with folate often result in negative data. However, these diseases develop very slowly, and during a test that lasts only a few years, endothelial cells or neurons are either already damaged, or the disease has progressed insufficiently for necrosis to have developed.

## Genetic Polymorphisms of Folate Metabolism

Serum folate and total homocysteine (tHcy) levels are influenced by both folate intake and genetic polymorphisms. There are several single nucleotide polymorphisms (SNPs) that affect folate metabolism, including the 5, 10‐methylenetertahydrofolate reductase (MTHFR) gene C677T. The presence of the C677T mutation in the MTHFR gene (rs1801133) has been regarded as a genetic risk factor for coronary artery diseases and neural tube defects (Li et al. 2016b). The frequency of the T allele of MTHFR is 36.9%, 41.7%, 58.6% and 8.1%, in Japan, the USA, Mexico and Indonesia, respectively (Sadewa et al. 2002). The authors also tested the following high frequency SNPs affecting folate and homocysteine metabolism (Hiraoka et al. 2004): MTHFR A1298C, CBS844ins68 (cystathionine‐β‐synthase), MS A2756G (methionine synthase) (Cai et al. 2014), MTRR A66G (methionine synthase reductase) (Zhi et al. 2016), and RFC‐1 G80A (reduced folate carrier‐1) (Li et al. 2016a). CBS844ins68 is the 8th exon insertion mutation and comprises 68 base pairs (Cai et al. 2014). However, these SNPs, except for MTHFR C677T, did not significantly affect serum Hcy, folate, vitamin B_6_ and vitamin B_12_ levels (Hiraoka et al. 2004). The frequencies of mutant homozygotes were lower in MTHFR A1298C (1.3%), MS A2756G (5.3%) and MTRR A66G (8.5%) than in MTHFR C677T (17.3%) and RFC‐1 A80G (18.6%), and thus may not affect the overall incidence of disease in large populations. In contrast, the TT genotype of MTHFR C677T showed significantly lower serum folate and higher serum Hcy than the CC and CT genotypes (Hiraoka et al. 2004). The TT genotype of the C677T polymorphism in MTHFR has been associated with CVD (Morita et al. 1998), dementia (Kageyama et al. 2008), osteoporosis (Gjesdal et al. 2007), depression (Bottiglieri 2005) and kidney diseases (Sakamoto et al. 2015). In addition, there are many risk SNPs of CVD, metabolic syndrome, and other chronic diseases (Kagawa 2012; Kagawa et al. 2012) that affect the MTHFR genotypes and must be considered for disease prevention and the reduction of medical costs.

Information obtained from the above studies on SNPs indicates that folate intake sufficient to reduce plasma Hcy to a minimum level of less than 7.0 μmol/L is required to reduce disease risk (Joint FAO/WHO Expert Consultation on Human Vitamin and Mineral Requirements 1998). In Japan, however, a recommendation for pregnant women to take a folate supplement was made by the Ministry of Health, Labor and Welfare only in 2000. The possible benefit of lowering plasma homocysteine through increased folate intake can be proven only by an intervention trial with folic acid supplementation in large populations. Using plasma homocysteine as a biomarker for folate adequacy can only be done on an individual basis after the possibility of a genetic mutation or an inadequate supply of vitamin B_6_ or vitamin B_12_ has been eliminated (Joint FAO/WHO Expert Consultation on Human Vitamin and Mineral Requirements 1998). The risk of hyperhomocysteinemia in subjects with the TT genotype of MTHFR C677T was successfully corrected by taking 400 μg/day of folate (Hiraoka et al. 2004), which is identical to the FAO/WHO and US recommended dietary allowance (RDA) of folate (Joint FAO/WHO Expert Consultation on Human Vitamin and Mineral Requirements 1998).

## Medical Cost Savings Resulting From Folic Acid Fortification

Folic acid supplementation of the expectant mother's diet should result in fewer NTDs among infants and ancillary savings in medical costs (Van Allen et al. 1993). A minimum dosage of folic acid of 0.8 mg/day and a maximum dosage of 5.0 mg/day was recommended (Van Allen et al. 1993), along with a well‐balanced, nutritious diet for all women who are at increased risk of having offspring with NTDs and who are planning a pregnancy. The results from economic evaluations demonstrate that folic acid fortification of food and preconception folic acid consumption are cost‐effective ways to reduce the incidence and prevalence of NTDs (Yi et al. 2011). The annual direct medical cost per patient was estimated to be €42 943 ($51 574 in 2003) for NTD and between €11 728 ($11 061 in 1993) to €54 270 ($65 177 in 2003) for spina bifida in the USA (Yi et al. 2011). The prevention of CVD and the medical cost savings expected from folic acid supplementation was also estimated (Hornberger 1998). Bayesian statistical and decision‐analytic techniques were used to estimate the cost–benefit and sample size of a placebo‐controlled trial of folate targeted to US citizens aged 35 to 84 years with elevated serum homocysteine levels. The main end point is event‐free survival (*i.e.*, survival without new ischemic heart disease or stroke) at 5 years. Because the screening and other costs associated with this study were small compared with the consequences of stroke, ischemic heart disease, or death, the increase in 5‐year event‐free survival with folate that should compel the use of folate is just 1.1% which was explained in detail **(**Hornberger 1998). The event‐free survival rate for the placebo and folate groups were assumed to lie, with 95% probability, between 83.7% and 87.7%, and 82.9% and 90.7%, respectively. Such a trial would provide an expected societal cost–benefit savings exceeding $11 billion within 15 years (Hornberger 1998).

In 1998**,** compulsory folic acid fortification was started in the US and Canada, and the FDA mandated that all enriched cereal grain products be fortified with folic acid at a level of 140 μg/100 g of cereal product (Ganji and Kafai 2006). The exact human test using ^13^C‐ and ^2^H double labeled folate fortified cereals established this fortification level (Pfeiffer et al. 1997). This fortification mandate is estimated to have reduced the annual number of US live‐birth spina bifida cases by 767 (Grosse et al. 2016). The present value of mean direct lifetime cost per infant with spina bifida is estimated to be $791 900, excluding caregiving costs. Folic acid fortification is estimated to reduce the present value of total direct costs for each year's birth cohort by $603 million above the cost of fortification (Grosse et al. 2016). These estimates of cost savings are larger than those previously reported, even using conservative assumptions.

Following the implementation of mandated folic acid fortification, a decline in stroke mortality was observed in the US between 1990 and 1997, and this decline accelerated in 1998 to 2002 in nearly all population strata, with an overall change from −0.3% (95%CI, −0.7 to 0.08) to −2.9 (95%CI, −3.5 to −2.3) per year (*P* = 0.0005) (Yang et al. 2006). The fall in stroke mortality in Canada averaged −1.0% (95%CI, −1.4 to −0.6) per year from 1990 to 1997 and accelerated to −5.4% (95%CI, −6.0 to −4.7) per year in 1998 to 2002 (*P* ≤ 0.0001). In contrast, the decline in stroke mortality in England and Wales did not change significantly between 1990 and 2002 (Yang et al. 2006). After the implementation of compulsory folic acid fortification, trends in serum folate, red blood cell (RBC) folate, and circulating total homocysteine concentrations in the United States were analyzed in 17 144 participants in the National Health and Nutrition Examination Surveys (Ganji and Kafai 2006). Overall, the geometric mean serum folate concentrations (nmol/L) were 12.1 ± 0.3, 30.2 ± 0.7, and 27.8 ± 0.5 in 1988–1994 (before fortification), 1999–2000 (after fortification), and 2001–2002 (after fortification), respectively. This means that the serum folate levels were 149.6% and 129.8% higher in the survey participants in 1999–2000 and 2001–2002, respectively, compared to in 1988–1994 (*P <* 0.0001). The RBC folate concentrations (nmol/L) were 391 ± 5.4, 618 ± 11.7, and 611 ± 9.3 in 1988–1994, 1999–2000, and 2001–2002, respectively. Age‐, sex‐, and race‐ethnicity‐adjusted tHcy declined from 9.5 μmol/L in 1988–1994 to 7.6 μmol /L in 1999–2000 and to 7.9 μmol/L in 2001–2002.

The greatest benefits from fortification were predicted in myocardial infarction prevention, with 88 172 cases averted per year in steady state condition after 5 years' folic acid fortification for a fortification level of 700 μg/100 g enriched grain (Bentley et al. 2009). This level of fortification, discussed by Dary (2009)**,** was also projected to avert 38 805 cases of colon cancer and 1423 cases of NTD. Compared with no fortification, all post‐fortification strategies provided Quality Adjusted Life Year (QALY) gains and cost savings for all subgroups, with predicted population benefits of 266 649 QALY gained and $3.6 billion saved in the long term by using a fortification level of 700 μg/100 g enriched grain.

## Outline of the Sakado Folate Project

The Japanese RDA is insufficient for persons with polymorphism in genes involved in folate metabolism and in aged persons with decreased folate bioavailability. The average folate intake in 2014 was 284 μg/day, which is higher than the Japanese RDA of 240 μg/day for adults (Ministry of Health, Labor and Welfare 2014). However, the folate intake of women aged 20–39 years was only 243–253 μg/day in 2014, which is much less than the RDA of 480 μg/day for pregnant women (Ministry of Health, Labor and Welfare 2014). There are three reports on the folate intake of Japanese pregnant women: 289 ± 150 μg/day (Mito et al. 2007), 336 ± 161 μg/day (Nakano and Ishii 2004) and 283 μg/day (Kondo et al. 2013, standard deviation not clear, the value of 2011). These values are much less than the RDA of 480 μg/day. The folate intake of 50% of the poor pregnant women was less than 260 μg/day (Onda R, reported in the Japanese Society of Nutrition and Dietetics, 2015). The folate intake higher than these levels among Japanese was quite effective in preventing cardiovascular diseases was established by Japan Collaborative Cohort Study on a total of 23 119 men and 35 611 women, age 40 to 79 years (Cui et al. 2010). Increased dietary folate intake from < 272 μg/day to > 536 μg/day resulted in 51% reduction of mortality for men from heart failure (*P <* 0.01) and that from ischemic heart diseases (down to 72%, *P <* 0.05), and for women ischemic heart diseases (down to 56% *P <* 0.01). These inverse associations did not change materially after adjustment for cardiovascular risk factors, so, folate intake of 243–253 μg/day by general Japanese is not enough to prevent cardiovascular diseases and thus more folate intake will proportionally reduce the medical costs.

Moreover, the bioavailability of folate is low in persons with genetic polymorphism (Hiraoka et al. 2004). About 15% of Japanese with the TT genotype of MTHFR C677T require 400 μg/day of folate to increase the serum folate level to that of CC and CT genotypes. Folate deficiency among persons with dementia was confirmed by meta‐analysis of 31 studies (Lopes da Silva et al. 2014) and of Japanese dementia studies (Kageyama et al. 2008). The risk of brain infarction is 3.4‐fold higher in persons with the TT type compared to persons with the CC type (Morita et al. 1998). The randomized controlled double blind test was performed to confirm the exact folate requirement of Japanese (Hiraoka et al. 2014).

**Figure 1:**
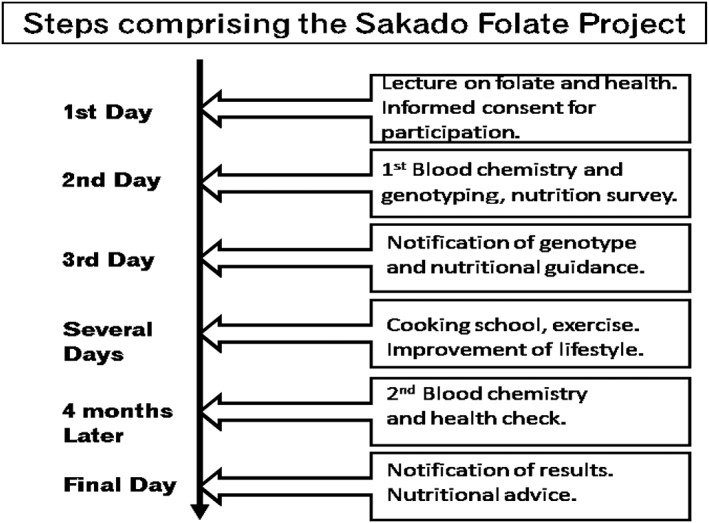
Steps comprising the Sakado Folate Project.

To prevent the above‐described diseases caused by folate deficiency, Sakado City in Japan made an agreement with Kagawa Nutrition University called the “Sakado Folate Project” in 2006. The Project offered participants a lecture, genotyping, blood analysis, nutrition surveys, and guidance according to the collected data (Fig. 1). The lecture was similar to that given in the Biomarkers of Nutrition for Development (BOND) project and was designed to provide evidence‐based advice to anyone with an interest in the role of folate, vitamin B_12_, and other micronutrients in health (Bailey et al. 2015). We provided people with an overview of available biomarkers (serum folate and homocysteine concentrations) and the interpretation of these biomarkers across a range of clinical and population‐based uses. We also explained the genetic polymorphisms involved in folate deficiency, and obtained written informed consent from participants in accordance with the instructions of the Declaration of Helsinki. The study procedures were approved by the Kagawa Nutrition University, Human Subjects and Genome Ethics Committee. There were 1101 participants (male: *n* = 345, female: *n* = 756) aged 61 ± 10 years. All participants completed a dietary intake analysis by means of a validated, brief self‐administered diet history questionnaire (DHQ‐L) (Sasaki 2004) containing questions about the consumption frequency of 56 foods and beverages commonly consumed by the Japanese population. Blood chemistry analysis included serum folate and tHcy concentration. The serum was isolated and stored at −80°C until analysis. Serum folate and vitamin B_12_ concentrations were measured at an external laboratory (SRL, Inc., Tokyo, Japan) using an Access 2 chemiluminescence enzyme immunoassay (Beckman Coulter, Inc., Carlsbad, *CA*). Serum tHcy concentration was determined by enzyme assay using an Alfressa Auto Hcy kit (Alfressa Pharma, Inc., Osaka, Japan) (Hiraoka et al. 2014). In order to genotype a large number of blood samples rapidly and at low cost, we developed an automated genotyping machine using a bead array in capillary tubes (Kagawa et al. 2010) and used this approach for MTHFR and other risk SNPs (Kagawa et al., 2012).

One month after obtaining the blood samples, the genotype was announced by the attending medical doctor to subjects who had agreed to know their genotypes, and nutritional and exercise guidance was provided by a registered dietitian. To supply enough folate easily, we developed “folate fortified rice” containing 26.7 mg folate, 187 mg thiamin, 66.7 mg vitamin B_6_ and 320 μg vitamin B_12_ per 100 g rice in collaboration with House‐wellness Foods Company. We also developed a folate fortified bread called Sakado Folate Bread (folic acid 340 ± 21 μg/100 g (215 ± 14.7 μg/slice/64.0 g bread) and succeeded in improving folate status and depressive states (Hiraoka et al. 2014). Commercially available Sakado Folate Bread contains 160 μg folic acid/slice.

**Figure 2:**
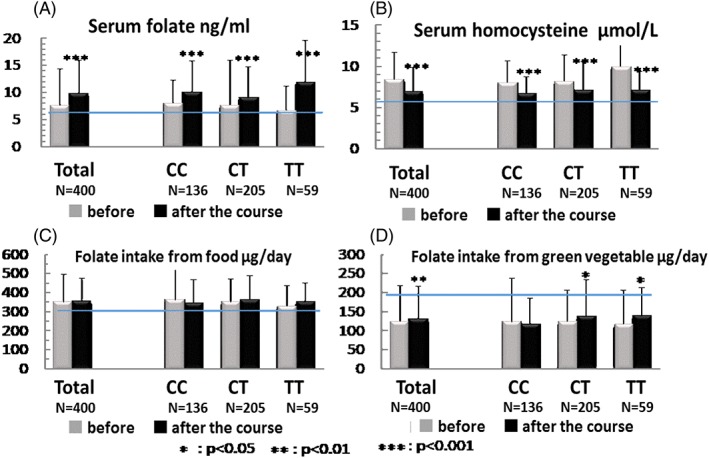
Effects of the Sakado Folate Project on serum folate and homocysteine, and the intake of dietary folate and green‐yellow vegetables, on individuals carrying three genotypes of MTHFR. (A) Serum folate concentration (ng/mL, y‐axis); the horizontal line indicates the standard (7 ng/mL). (B) Serum homocysteine (μmol/mL, y‐axis), and the horizontal line indicates the standard (6 μg/mL). (C) Intake of dietary folate (μg/day, y‐axis), and the horizontal line indicates the standard (300 μg/day). (D) Intake of dietary green‐yellow vegetables (μg/day, y‐axis), and the horizontal line indicates the standard (200 μg/day).

Serum folate concentrations were significantly increased and serum tHcy concentrations decreased at 4 months after the onset of this intervention by the Project (Fig. 2). The horizontal lines in panel A (serum folate), B (serum homocysteine), C (dietary folate intake) and D (dietary green‐yellow vegetables) indicate the standard values (7 ng/mL), (6 μg/mL), (300 μg/day) and (200 μg/day), respectively. In individuals with the TT genotype, these changes were particularly marked and intake of ‘green vegetables’, which are rich in folate, was increased in the TT genotype participants. After the instructional Project, 86% of the subjects consumed folic acid fortified food, of which 42% consumed folate‐fortified rice every day.

The most important aspects of the Project were the health education of about 101 000 citizens due to the efforts of volunteers and wider consumption of folate‐fortified food, especially folate‐fortified rice. The above‐mentioned BOND project (Bailey et al. 2015) was designed to easily explain the role of folate and vitamin B_12_ to the general public by volunteer nutritionists. In Sakado Folate Project, we also obtained help of 125 nutrition advisors (nutrition mates) who both visited people's neighborhoods and worked in numerous facilities called “Terakoya”. BOND project was similar in their guidance principle, but the implementation of Sakado Folate Project using nutritional advisors, “Terakoya” and other measures were quite different from those of BOND project. According to the official report prepared by Sakado City on the nutritional behavior of its citizens, after the start of this Project 80% of the population knew about the importance of folic acid, 90% tried to consume more vegetables, and 73% wanted to obtain health advice. The population of Sakado City was only about 101 000 in 2015 and there were 693 births in 2015; consequently, a decrease in neural tube defects could not be confirmed. However, the birth rate of very low birth weight infants (body weight < 1500 g) decreased from 7.8 (1998–2002) to 6.1 (2006–2010) per thousand births after the Project. The total fertility rate in the first period was 1.13 and second period was 1.20 (Ministry of Internal Affairs. Statics Bureau of Japanese Government, 1990a,b).

There are five reasons for the overall success of the Sakado Folate Project.

1. Effective organization of volunteers recommended by the government to promote a healthy diet.
2. The invention of a rapid and inexpensive genetic polymorphism analyzer.
3. The involvement of a well‐trained registered dietician in the nutritional survey who was available throughout the project to give advice.
4. The cooperation of the local government and the mayor of Sakado City.
5. The development of two folate‐fortified foods: Sakado Folate Rice and Sakado Folate Bread.

## Medical Cost Savings Resulting from the Sakado Folate Project

**Figure 3:**
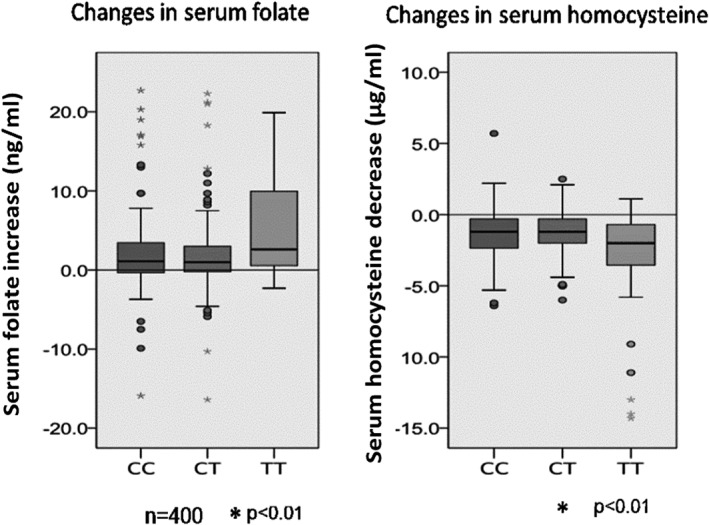
Effects of genome notification on changes in serum folate and serum homocysteine in individuals carrying three different genotypes of MTHFR (CC, CT and TT). Bars represent the means ± SD.

We verified the effect of personalized nutrition intervention on the folate status of participants; this, together with genotype notification, was effective in motivating individuals to change their dietary habits and improve their folate intake (Fig. 3). Thus, the subjects with the most risky TT genotype showed the highest increase in serum folate and the largest decrease in serum homocysteine (Figs 2, 3).

**Figure 4:**
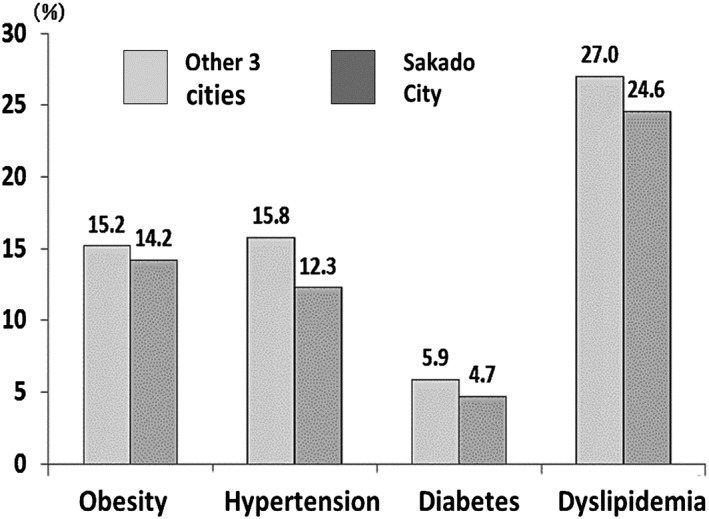
Prevalence of obesity, hypertension, diabetes and dyslipidemia among the population in Sakado City and three other cities in Saitama Prefecture (Saitama Prefecture Local Government 2011). The ordinate indicates prevalence in percentage. The prevalence in Sakado City was significantly fewer than that in other three cities in men (*P =* 0.04).

Both the prevalence of disease and the medical costs of subjects participating in the Project, and of the general Sakado population, were reduced. A nutrition survey conducted by the Saitama Prefecture Department of Health in 2011 revealed that the prevalence (% of adult population) of obesity, hypertension, type 2 diabetes and dyslipidemia diagnosed by physicians was significantly lower than that in the males of three other cities (Fig. 4). The data on Figure 4 are based on adult population in 2011. The male population showed significantly lower prevalence of these diseases than the other three cities (*P =* 0.04), but female population did not reach significant level. The average ages of males and females in Sakado city in 2011 were 42.4 and 44.4, respectively, while those of three cities (Yoshikawa, Asaka and Okegawa cities) on average were younger; 41.4 and 43.1 (Ministry of Internal Affairs. Statics Bureau of Japanese Government, 1990a,b).

**Figure 5:**
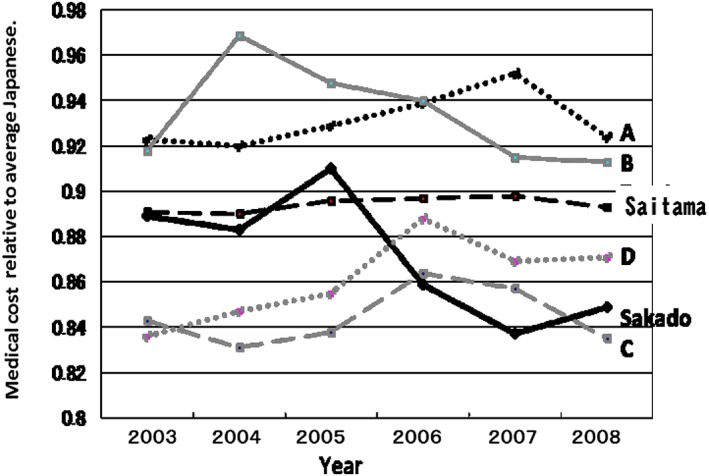
Trend in relative medical costs in Sakado City compared with four cities in Saitama Prefecture. The abscissa shows the year and the ordinate indicates the medical costs incurred by each city relative to the average total medical costs in Japan (=1.00) taken from the home page of the Ministry of Health, Labor and Welfare (http://www.mhlw.go.jp/topics/bukyoku/hoken/iryomap/). The relative decrease of medical costs from 2003–2005 to 2006–2008 in Sakado City plotted in Figure 5 is statistically significant (*P =* 0.0459, using Wilcoxon rank sum test). Black thick line: Sakado City, black broken line: average for Saitama Prefecture. Black dotted line: city A, gray line: city B, gray broken line: city C, and gray dotted line: city D.

This was reflected in the relative decrease in medical costs in Sakado City immediately after the start of the Project in 2006 (Fig. 5). The relative decrease of medical costs from 2003–2005 to 2006–2008 in Sakado City plotted in Figure 5 is statistically significant (*P =* 0.0459, using Wilcoxon rank sum test). The actual medical costs of National Health Insurance per person was ¥303 900 in 2006–2007 compared with those of ¥290 399 in 2001–2005 in Sakado City. The increment ¥13 501 was the smallest among all cities in Saitama Prefecture. The abscissa in Figure 5 is the year and the ordinate indicates the medical costs for each of the four cities being compared relative to the average total medical costs in Japan (1.00). A few years may be necessary for this Project to expect a reduction in the significant amount of the medical expenditure. However, in the large population of Sakado city, similar to United States as soon as the serum folate elevation, rapid decrease in stroke took place within a year (Yang et al. 2006), because the increase of serum folate and decrease of serum homocysteine takes place only within a few weeks (Hiraoka et al. 2004, 2014). Moreover, rapid preventive effects of folate on cardiac infarction was reported in detail (Moens et al. 2008). In addition, the subjects of the Project in 2006 and 2007 were mainly local health and nutrition leaders to teach citizens rapidly. Since there were no rapid demographic change in Sakado city (Ministry of Internal Affairs. Statics Bureau of Japanese Government, 1990a,b), the rapid reduction in the medical expense is attributed to the Project. We should show the values to the year 2014 or 2015 in Figure 5, however, unfortunately, there was a large reform of the medical insurance system during 2008, which is Comprehensive Reform of Social Security: Advanced Elderly Medical Service System (Medical care for the elderly over 75 years old) (Ministry of Health, Labor and Welfare 1990), so we have to discontinue the graph at 2008. Although the total medical cost were changed, in 2014, that of Sakado city (average age: male 42.8, female 44.7) was 95.7% of that of all cities in Saitama (average age: male 42.3, female 44.3), and 92.5% of that of neighboring Kawagoe city (average age: male 42.6, female 44.7).

The average medical costs saved per person per month (during May and October before the project from 2003 to 2005 minus those after the project from 2006 to 2008) after the Project for cardiac infarct, cerebrovascular disease, dementia and osteoporosis were ¥364–297 = −¥67, ¥420‐¥409 = −¥11, ¥85‐¥47 = −¥38 and ¥230‐ ¥222 = −¥8, respectively. These data were based on the automatic computer output of Japanese Health Care Insurance System of Sakado city, and the medical costs were analyzed by experts of medical epidemiology. However, the results for average citizens shown in Figures 4 and 5 do not represent the direct effects of folate intake because we did not measure the serum folate levels of all citizens.

The relationship between the costs and the changes in serum folate level was used to estimate the medical cost savings (Bentley et al. 2009). The proportional association between the changes in serum/plasma folate and the intake of folate in the diet based on information from several studies reporting these two parameters were calculated using a linear equation that describes their association (Quinlivan and Gregory 2007): change in serum folate (mg/L) = [intake of folic acid as DFE (μg/day) × 0.0145] + 0.132 (Dary 2009). The formula considers the equivalence of folic acid in terms of DFE based on the approximately 70% higher bioavailability of monoglutamyl pteridine over dietary sources of folate. Table 1 summarizes the changes in serum folate of three genotypes of MTHFR before and after the Project, with the intake of folate being expressed in terms of synthetic folate (monoglutamyl pteridine). The serum folate concentration in the 1101 subjects increased from 7.68 ng/mL to 9.92 ng/mL, a change of 2.24 ng/mL. However, the subjects notified of carrying the TT genotype (*n* = 191) showed serum folate concertation increased 178% from 6.87 (17.4 nmol/L) to 12.22 (22.5 nmol/L). nmol/L). The calculated medical costs saved per person was ¥1,669 for a total of ¥1,784,042 per year (Sakado City Local Government 2015 **)** according to the relationship between folate intake and medical cost reported (Bentley et al. 2009), if the relationship proposed by Bentley is applicable to the people of Sakado.

**Table 1:** Estimated medical cost savings resulting from folate intake by the residents of Sakado City

Genotypes	Serum folate (ng/mL)	Changes in synthetic folate intake (μg/d)	Medical costs saved per year/person	Total medical costs saved per year
	Changes			
CC (*n* = 357)	2.11	80.39	¥1644	¥586 900
CT (*n* = 553)	1.46	54.00	¥1392	¥769 766
TT (*n* = 191)	5.34	211.47	¥2238	¥427 377
Total (*n* = 1101)	2.24	85.58	¥1669	¥1 784 042

**Figure 6:**
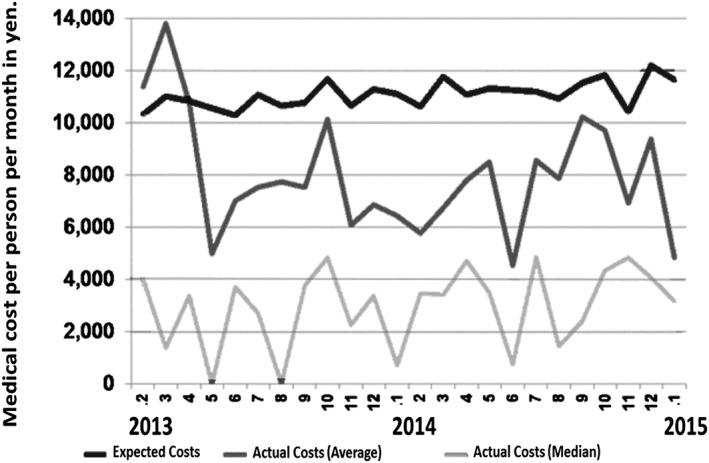
Expected medical costs and actual medical costs of subjects participating in the Sakado Folate Project. The abscissa shows the year and the ordinate indicates the medical costs per person per month in yen. Black line: expected costs of citizens in Sakado City, Gray line: average of actual costs of the subjects participating in the Project, Light gray line: median of the actual costs of the subjects participating in the Project.

However, these estimated medical cost savings were limited to coronary infarction, colon cancer and spina bifida (Bentley 2009), and therefore we measured the medical cost savings of the subjects participating in the Project (Fig. 6). To do this, we compared the average medical costs of Sakado citizens (expected costs: ¥10 276 to ¥12 196; average: ¥11 076 per person per month) with those of participants in the Project. The average medical costs of the participants fluctuated greatly, between ¥4518 to ¥13 793, (average: ¥7958 per person per month), due to the small number of participants, and this caused large skewness of the frequency distribution of personal medical cost, because some rare medical treatment costs extremely high and affects the average value. Thus, the median (¥0 to ¥4830, average: ¥2960, per person per month) clearly shows medical cost savings of ¥8116 per person per month (a cost savings of 26.7% compared to the control citizens) attributed to the Sakado Folate Project (Fig. 6). According to the medical cost calculation system there are always some delay in the exact sum up. So the curve is an expected value considering the recent medical costs, not the exact medical cost.

Finally, mandatory folic acid fortification (Bentley et al. 2009) is obviously more cost‐effective than the Project, because in addition to the cost of folic acid fortification, the Project incurred other costs per participant, such as personnel expenses for education on nutrition (¥1500 per person per group lecture), nutritional surveys (for example, the FFQ type cost ¥1000 per person), genotyping (about ¥500 per person), and general biochemical analyses (¥110 = 11 analyte per person). Moreover, following supplementation, serum folate levels increased from 12.1 to 30.2 nmol/L (an increase of 149.6%) in the USA (Ganji and Kafai 2006), but only from 17.4 to 22.5 nmol/L (an increase of 129% averaged between 2006 and 2012) in Sakado. Country‐specific differences in the medical care costs for treating patients with each disease, as well as personnel expenses, fluctuations in the exchange rate between the American dollar and the Japanese yen, and the numerous assumptions required, together made it difficult to calculate the exact cost benefits of the Project compared with that of mandatory folic acid fortification.

## Conclusion

In summary, folic acid fortification was effective in preventing not only NTDs but also other diseases, including CVD, dementia, kidney diseases and osteoporosis, irrespective of genetic polymorphisms such as the MTHFR TT genotype. The medical costs saved were dependent on the disease involved, the projects implemented in each country, and the economic evaluation methods used. The genotyping was effective in high risk approach with increased motivation (Figs 2, 3)**.** Genome notification played an important role in the Sakado Folate Project and was effective in motivating folate intake, especially in participants with the TT genotype, yet the increase in serum folate (129%) was less than that observed following compulsory folic acid fortification in the USA (149.6%). Evaluation showed that compulsory folic acid fortification is more cost‐effective than the genotype‐based educational approach used in the Sakado Folate Project. In conclusion, compulsory folic acid fortification is the best method to prevent disease and reduce medical costs.

## Disclosures

None.
